# Plain-to-clear speech video conversion for enhanced intelligibility

**DOI:** 10.1007/s10772-023-10018-z

**Published:** 2023-01-28

**Authors:** Shubam Sachdeva, Haoyao Ruan, Ghassan Hamarneh, Dawn M. Behne, Allard Jongman, Joan A. Sereno, Yue Wang

**Affiliations:** 1 Language and Brain Lab, Department of Linguistics, Simon Fraser University, Burnaby, BC Canada; 2 Medical Image, Analysis Research Group, School of Computing Science, Simon Fraser University, Burnaby, BC Canada; 3 NTNU Speech Lab, Department of Psychology, Norwegian University of Science and Technology, Trondheim, Norway; 4 KU Phonetics and Psycholinguistics Lab, Department of Linguistics, University of Kansas, Lawrence, KS USA

**Keywords:** Video speech synthesis, Speech style, Intelligibility, AI lip reading, Speech enhancement

## Abstract

Clearly articulated speech, relative to plain-style speech, has been shown to improve intelligibility. We examine if visible speech cues in video only can be systematically modified to enhance clear-speech visual features and improve intelligibility. We extract clear-speech visual features of English words varying in vowels produced by multiple male and female talkers. Via a frame-by-frame image-warping based video generation method with a controllable parameter (displacement factor), we apply the extracted clear-speech visual features to videos of plain speech to synthesize clear speech videos. We evaluate the generated videos using a robust, state of the art AI Lip Reader as well as human intelligibility testing. The contributions of this study are: (1) we successfully extract relevant visual cues for video modifications across speech styles, and have achieved enhanced intelligibility for AI; (2) this work suggests that universal talker-independent clear-speech features may be utilized to modify any talker’s visual speech style; (3) we introduce “displacement factor” as a way of systematically scaling the magnitude of displacement modifications between speech styles; and (4) the high definition generated videos make them ideal candidates for human-centric intelligibility and perceptual training studies.

## Introduction

This study examines whether visible speech cues in video can be systematically modified to enhance speech features and improve intelligibility. To this end, we capture visual cues in clear (hyper-articulated) speech produced by multiple talkers and then leverage these cues to modify the visual plain (conversational) speech by novel talkers.

### Background

Naturally produced clear speech has been shown to be as much as 7–38% more intelligible than its plain counterpart (Ferguson & Kewley-Port, 2002; Ferguson & Quené, 2014; Maniwa et al., 2008; Payton et al., 1994; Redmon et al., 2020; Uchanski et al., 1996). Clear speech can also improve intelligibility in visual (facial) speech perception (Gagné et al., 1994, 2002; Helfer, 1997; Lander & Capek, 2013; Van Engen et al., 2014). The increase in intelligibility can be attributed to enhanced auditory and visual cues occurring in clear speech relative to plain speech. In the auditory signal, clear speech involves changes in pitch, duration, and spectral dynamics (Cooke & Lu, 2010; Ferguson & Kewley-Port, 2002, 2007; Hazan & Baker, 2011; Kim & Davis, 2014a; Krause & Braida, 2004; Leung et al., 2016; Maniwa et al., 2009; Smiljanić & Bradlow, 2005). In clear visual speech, key articulatory movements such as lip stretching and jaw lowering are exaggerated (Kim & Davis, 2014b; Kim et al., 2011; Tang et al., 2015; Tasko & Greilick, 2010).

Specifically relevant for the current study is Tang et al. (2015), which examined the videos of talkers’ faces in the production of English words containing tense vowels (/i, ɑ, u/) and lax vowels (/ɪ, ʌ, ʊ/), using computer-vision and image processing techniques. The results show that clear compared to plain speech productions involve longer and greater vertical lip stretch and jaw displacement across vowels, greater horizontal lip stretch for front unrounded vowels, and greater degree of lip rounding for rounded vowels. Further intelligibility research (Redmon et al., 2020), however, reveals that these visual cues are predictive of a clear-speech advantage for tense vowels and not lax vowels, presumably because lip stretching in clear speech deviates from lax vowel features and thus decreases tense-lax vowel distinctions (Leung et al., 2016). Indeed, clear-speech modifications are claimed to involve a trade-off between “contrast enhancement” and “maintenance of phonemic norms” in intelligibility benefit (Moon & Lindblom, 1994; Ohala, 1995; Smiljanić et al., 2021). Excessive exaggerations or modifications incompatible with sound-inherent cues (e.g., greater horizontal lip-stretching of lax vowel /ɪ/) may obscure visual distinctiveness between sounds (e.g., /i/-/ɪ/) and inhibit intelligibility (Redmon et al., 2020). These patterns indicate that clear-speech modifications need to be within category boundaries in order to benefit speech intelligibility.

Research has also shown that individual talkers vary in their implementation of clear speech. For instance, modifications by talkers who do not reach a threshold of contrast may not provide clear-speech benefits in intelligibility (Ferguson & Kewley-Port, 2007; Smiljanic & Bradlow, 2011). Clear-speech effects have also been shown to be greater for female than male talkers (Ferguson, 2004, 2012). Additionally, talker-specific visual features may be encoded with phonetically relevant visual speech information, demanding additional cognitive resources to extract speech-related cues and thus hindering intelligibility (Yakel et al., 2000). It is therefore essential to disentangle how talker-specific and talker-universal clear-speech features affect intelligibility differently.

These clear speech influencing factors can be systematically assessed by computationally enhancing visual speech cues in video. Enhancing visual speech cues to generate clear speech may have significant benefits, for instance, in adverse listening environments, for language learners or hearing-impaired populations, or for automatic Lip Readers (Martinez et al., 2020). While most recent computer vision research on audio-visual speech enhancement includes visual information in addition to auditory information as input (Ideli et al., 2019; Sadeghi et al., 2020), output manipulations have typically been limited to enhanced auditory speech only. For example, Morrone et al. (2019) use facial landmarks as input to enhance auditory speech, and their success indicates that facial landmarks and movement in a video are effective features for auditory speech enhancement. In the work by Hou et al. (2018), lips were synthesized, but only as a “by-product” of their encoder-decoder AVDCNN network, and it is not known whether their synthesized lips result in increased intelligibility. With the aim of training listeners to perceive visual speech, Massaro and Light (2004) showed that adding visual cues particular to the phonemes and increasing the duration of productions helped hearing-impaired children in learning to recognize words. However, none of these studies translate plain speech to clear speech to improve intelligibility of enhanced videos, varying the magnitude of the visual manipulations, the topic of the current research.

### Hypotheses

To the best of our knowledge, our research is the first to test generating more intelligible speech videos by transferring video features from clear speech training data to plain videos of novel talkers. Our goal is to synthesize clear speech videos from plain speech videos by adding visual cues from clear speech and to test how effective these visual cues are. To assess the contribution of talker-specific information, we modify videos within the same talker and based on an average across talkers. Additionally, improvement in intelligibility due to our proposed enhanced video synthesis is tested by AI as well as by human participants by presenting the synthesized videos for identification to assess to what extent extreme visual changes will help intelligibility. Finally, we systematically vary the amount of visual displacement, based on the observed clear speech visual changes. Considering the beneficial factors of clear speech reviewed above, we hypothesize the following:

1. Synthetically enriching plain (conversational) videos with clear video features may improve intelligibility of tense-vowel words but not lax-vowel words, based on previous results of intelligibility of natural clear speech.
2. Modified videos based on clear-speech visual features averaged across talkers (average target) may improve intelligibility more than those based on a single talker (same-talker target). Our assumption is that visual features averaged across multiple talkers may overcome talker-specific idiosyncrasies (e.g., gender) and may thus more likely extract universal clear-speech visual features.
3. The increase in intelligibility of the synthesized enriched plain videos may be based on the extent to which the clear features are added to the plain videos. We control the extent via a displacement factor (DF) parameter (DF1, DF2, DF3). Visual modifications at a level equivalent to natural clear speech enhancement (e.g., DF1) are expected to improve intelligibility, more extreme visual modifications (e.g., DF2) may further improve intelligibility, and excessive modifications (e.g., DF3) may violate certain constraints characterizing sound categories and may thus inhibit intelligibility.
4. In intelligibility tests, we present videos containing modified visual speech cues (without audio). AI may outperform human participants (i.e., AI may achieve higher word recognition accuracy). This is expected due to the difference in task complexity for AI versus a human perceiver. For AI, classification is based on a finite number of features associated with clear speech and with much more restricted alternative recognition choices, whereas human perceivers may choose from a much wider set of alternatives and may be distracted by additional (facial, articulatory, or attentional) cues.

## Materials

The audio–video materials used in this study to generate the initial clear and plain tokens include multiple productions of six English words derived from three tense-lax vowel pairs /i-ɪ/, /ɑ-ʌ/, /u-ʊ/. These vowels were embedded in a monosyllabic /kVd/ context that yields the English words “keyed”, “kid”, “cod”, “cud”, “cooed”, and “could”.

Eighteen native English talkers (10 females, 8 males), born and raised in Western Canada, produced the target words both as plain (conversational) and as clear speech, where the speech styles were elicited using a simulated interactive computer speech recognition program established previously (Maniwa et al., 2009; Redmon et al., 2020; Tang et al., 2015). In this program, a talker was instructed to produce a target word naturally, as if in casual conversation (thus eliciting plain style productions). Then, the program would “guess” and display a “guessed” word on the screen and instruct the talker to repeat the word as clearly as possible for any incorrect “guess” (thus eliciting clear style productions). Multiple repetitions of each word were elicited based on different “guesses”. In total, each talker contributed 90 plain productions (6 words × 15 repetitions) and 72 clear productions (6 words × 12 repetitions).

The audio–video recordings were acquired in a sound-attenuated booth in the Language and Brain Lab at Simon Fraser University. Front-view videos were captured with a Canon Vixia HF30 camera at a recording rate of 29 frames per second with 1920 × 1080 pixel per frame. These videos were temporally segmented at the word level, with each clip 4 s long to ensure that both mouth opening and closing were captured. The videos were then segmented at the phoneme level and the following visual measurements were collected as they correspond to the inherent articulatory features of the three vowel pairs (Table 1): vertical lip stretching, horizontal lip stretching, jaw lowering, and lip roundness. For more details on the data collection, video segmentation, and calculation of these specific features, we refer the reader to Tang et al. (2015).

**Table 1:**
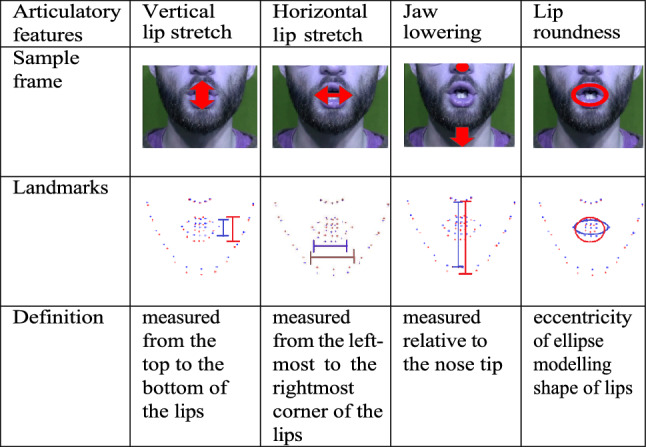
Visual cues used in the study

The blue landmarks are obtained from the mid-vowel frame of the plain (blue) video and the red landmarks are obtained from the mid-vowel frame of the clear (red) video. The “Landmarks” row shows how clear (red) landmarks have more stretch or eccentricity than plain (blue) landmarks

### Visual cues

Visual speech cues in the videos were then examined. As in Tang et al. (2015), going from plain to clear speech, the talkers change visual articulatory cues. These cues differ in magnitude across talkers but a consistent trend can be observed. For the three vowel pairs in question, similar to Tang et al. (2015), we examine the vertical and horizontal stretch of the lips, the lowering of the jaw and the roundness of the lips. In addition to these cues, we also add an area measurement of the lips as a measure of mouth opening.

**Fig. 1:**
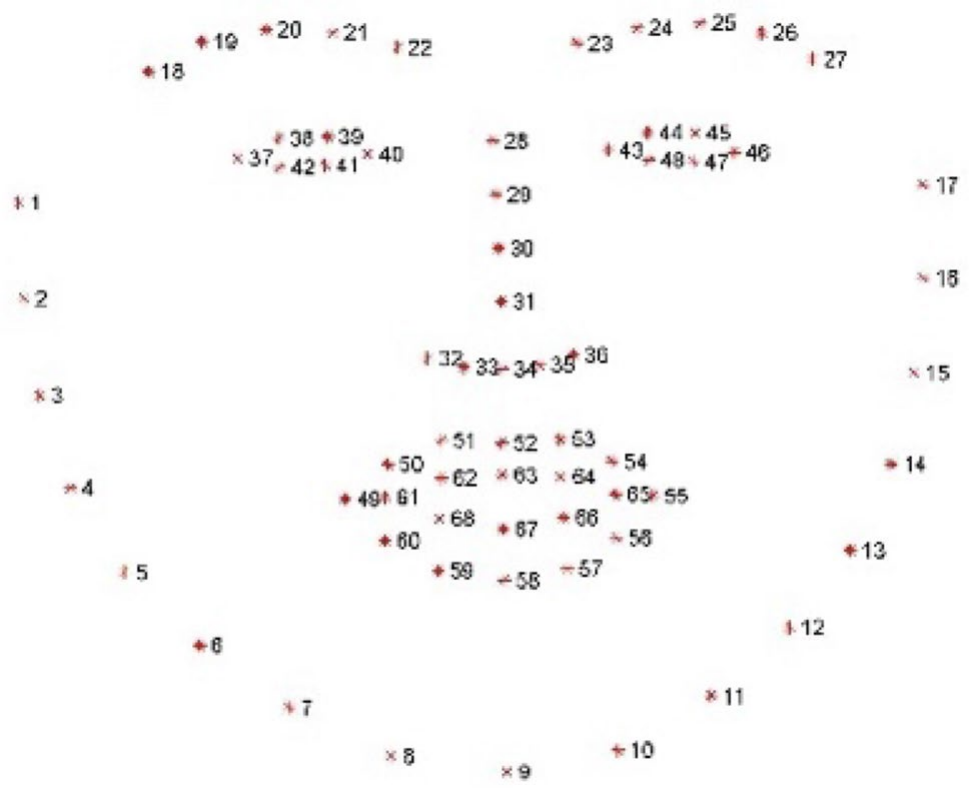
The 68 landmark positions on a face

To obtain these measurements, we first detect the face in each frame of the video using the dlib face detector which is one of the most utilized face detection libraries (King, 2009). It uses a pretrained Histogram of Oriented Gradients (HOG) to extract a 128-dimension vector followed by a Linear Support Vector Machine (SVM) to detect faces. Then, 68 facial landmarks (as shown in Fig. 1) on the detected face are extracted using the Style Aggregated Network (SAN) landmark detection method (Dong et al., 2018). The SAN network takes an image and generates multiple images in various styles using a Generative Adversarial Network (GAN) module. These generated images are then used to predict the facial landmarks. An averaged position of each of the landmarks across all images is output for increased robustness.

In the current study, we were specifically interested in the visual cues contributed by the lips (landmarks 48–68) and jaw (landmarks 6–12). We use $l_{k}$ to denote the *k*th landmark. Using these landmarks, we define the following visual measures (also shown in Table 1):

- *Vertical Stretch* of the lips is defined as the stretching produced by the lips measured from the top of the lips to the bottom, i.e., $V_{stretch} = \left| {l_{52} - l_{58} } \right|$*.*
- *Horizontal Stretch* of the lips is defined as the stretching produced by the lips measured from the left most corner to the lips to the right most, i.e.*,* $H_{stretch} = \left| {l_{55} - l_{49} } \right|$.
- *Jaw-Lowering* is defined as the downward movement of the jaw ($l_{9}$) relative to the tip of the nose ($l_{34}$). The nose tip reference is important in order to only consider jaw lowering due to mouth opening and ignore any jaw lowering due to moving the face down without opening the mouth (e.g. nodding). $Jaw_{lowering} = \left| {l_{9} - l_{34} } \right|$.
- *Roundness* of the lips. We model the shape of lips as an ellipse and use the eccentricity $e$ as a measure of roundness, where $e = \sqrt {1 - \frac{b}{a}}$, and $b$ is the minor axis of the approximating ellipse and is given by $b = {\text{minimum}}\left( {V_{stretch} ,H_{stretch} } \right)$ and $a$ is the major axis, $a = {\text{maximum}}\left( {V_{stretch} ,H_{stretch} } \right)$. Note that $0 < e < 1$, where $e = 0$ indicates the lips are fully rounded, i.e., $V_{stretch} = H_{stretch}$, whereas $e = 1$ indicates the lips are closed when $V_{stretch} = 0$.
- *Area* of the lips is defined as the area of the approximating ellipse and is given by $Area = \frac{1}{4}{\uppi }V_{stretch} H_{stretch}$.

## Clear speech synthesis methodology

### Overview

**Fig. 2:**
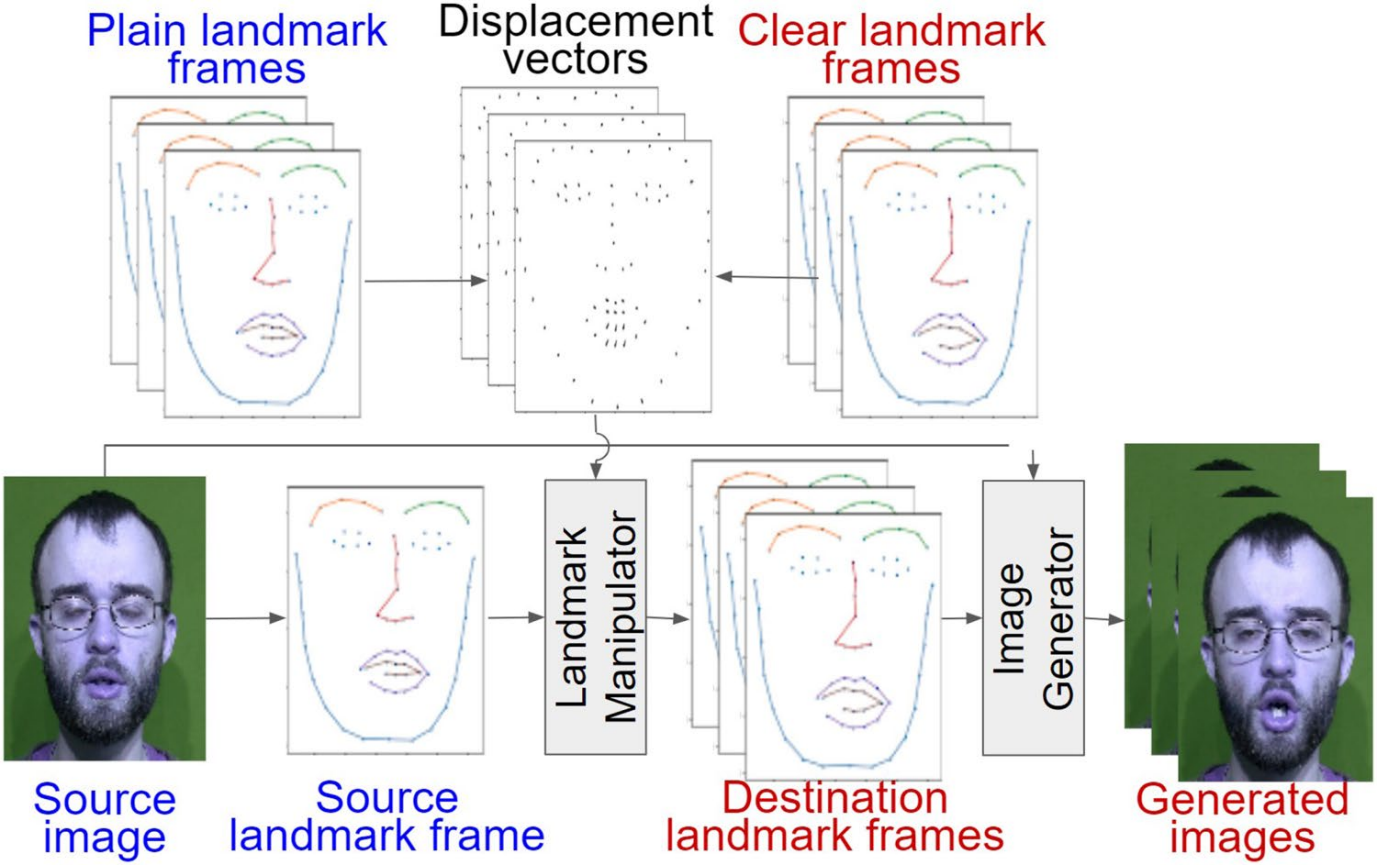
The overall image-generation pipeline. First, we extract facial landmarks from an image. A displacement vector is calculated as the difference between the landmarks in plain (blue) and clear (red) landmark frames. These vectors are then added to the source landmark frame of the plain-speech token in the Landmark Manipulator. Next, the generated landmark frames and the original image are passed into the Image Generator to produce the final synthesized video (series of images)

Our goal was to synthesize clear speech videos from plain speech videos by ‘adding’ visual cues particular to clear speech to the vowel in the word being spoken. We treat this video synthesis task as a frame-by-frame generation with temporal consistency (Fig. 2).

Following landmark normalization (Sect. 3.2), we apply a landmark manipulator (Sect. 3.3) to displace the landmark positions in a way that captures clear-speech articulatory movements. Landmark displacement modifications were implemented based on clear-speech cues extracted from the ground truth clear productions averaged across talkers. We also extract the displacements based on the clear-speech cues derived from the ground truth clear productions by the same talker in the plain-speech source (Sect. 3.4). A displacement vector is calculated as the difference between the landmark coordinates in plain and clear frames. The video synthesis operation is controlled using a tuning scaling ‘displacement factor’ (DF) that varies the magnitude of the added clear speech visual cues to the plain speech (Sect. 3.7). The modified landmarks and the source image are then used to generate the final image. An image generator (Sect. 3.8) repeats this process over all frames of the plain video to generate the synthesized clear speech video. Since, across plain and clear styles, videos vary not only in articulatory features but also in duration, we also address the temporal alignments (Sect. 3.5). Figure 2 shows the video generation computational pipeline.

### Landmark spatial normalization

Prior to processing the landmarks, we remove any captured movements due to head tilting or varying proximity of the talkers to the camera by applying a similarity spatial transformation. The landmarks are first centered around the tip of the nose. Then, face orientation is normalized by rotating the face such that the line (L) joining the ‘nose tip’ and the ‘mid-point between the two eyes’ points vertically upwards (positive y-axis). For scale normalization, the face is scaled to make the line L of unit length.

### Landmark manipulator

The landmark manipulator receives a single frame, along with its detected facial landmarks, that corresponds to the midpoint of the vowel production of a novel plain speech video. Given a training set (Sect. 2) of talkers uttering words in both plain and clear speech, we quantify how the landmarks’ positions change (displacements) from each frame depicting plain to the corresponding frame depicting clear speech. We then obtain visual cue ratios between each reconstructed frame and the mid vowel frame of the plain speech video. These ratios, when multiplied by the input mid vowel frame, give us the destination landmarks true to the input mid vowel landmark frame. This means that landmark frames are calculated using the landmarks and the displacements. This is done on a word-by-word basis. For instance, if the word in question is a plain speech “could”, only the features corresponding to the clear video of “could” from the same talker are used. See Algorithm 1 (Appendix 1) for details.

### Modification targets

Once we detect the facial landmarks from the plain-speech frame, we manipulate these landmarks to mimic how they appear in the clear-speech frame. We test two ways of modifying the targets:

1. *Same-talker target* Since the ground truth dataset contains a set of clear videos corresponding to the plain (conversational) videos from the same talker, we use the landmarks of the plain video (${\text{plain}}_{{\text{i}}}$) as the source plain landmarks. The corresponding ground truth clear video ($clear_{i}$) is used as the target clear video and its landmarks serve as the target clear landmarks. Therefore, clear-speech cues are extracted from the same talker. The advantage of the “same-talker target” approach is that it can capture all the idiosyncratic talker-specific differences between plain and clear landmarks from the same talker.
2. *Average target* Our second approach uses averaged clear-speech cues across different talkers as modification targets. We include all the available plain-speech videos and the corresponding clear videos for the same word across 14 talkers as training data (excluding four talkers’ videos held out as test data). Then, we learn the plain-to-clear changes for each of the plain and clear pairs from one talker and subsequently average the changes across talkers to apply to ${\varvec{plain}}_{{\varvec{i}}}$ and get as close as possible to ${\varvec{clear}}_{{\varvec{i}}}$. See Algorithm 2 (Appendix 1). Given that all destination faces are normalized for rotation and scale (Sect. 3.2) to align to the source face, we apply equal weighting to all the pairs. The advantage of the “average target” approach is that it can exclude talker-specific, idiosyncratic changes that do not necessarily pertain to clear speech. Further, in practice, the clear speech of a specific talker may not always be available when a synthesized one is desired.

### Handling duration discrepancy

As the duration of the target words changes with speech style (Leung et al., 2016; Tang et al., 2015), the number of frames may differ between natural plain speech videos and natural clear speech videos. Since the displacements are calculated in a one-to-one manner between frames of plain and clear speech, an equal number of landmark frames is required for both speech styles. To address this, we fit a degree 3 polynomial curve to the changing (over time) position of each of the 68 landmarks. The curves are fit to the x and y components of the landmarks separately, giving us 136 such curves. These continuous curves allow us to sample a landmark’s position at any sub-frame resolution. For instance, if there are 12 frames in the natural clear speech video and only 9 in the natural plain version, we sample the plain speech curves at 12 equidistant points to match the number of frames of the natural clear video in order to calculate the plain-to-clear displacement frame-by-frame. Then, to acquire 12 frames for the generated clear speech video from the 9-frame natural plain speech video, three adjacent frames in the middle of vowel portion were duplicated. Thus, the generated clear speech video involves more frames than, yet with the same frame rate of, the original plain speech video to match the lengthened duration in clear speech.

### Landmark smoothing

#### Temporal landmark smoothing

**Fig. 3:**
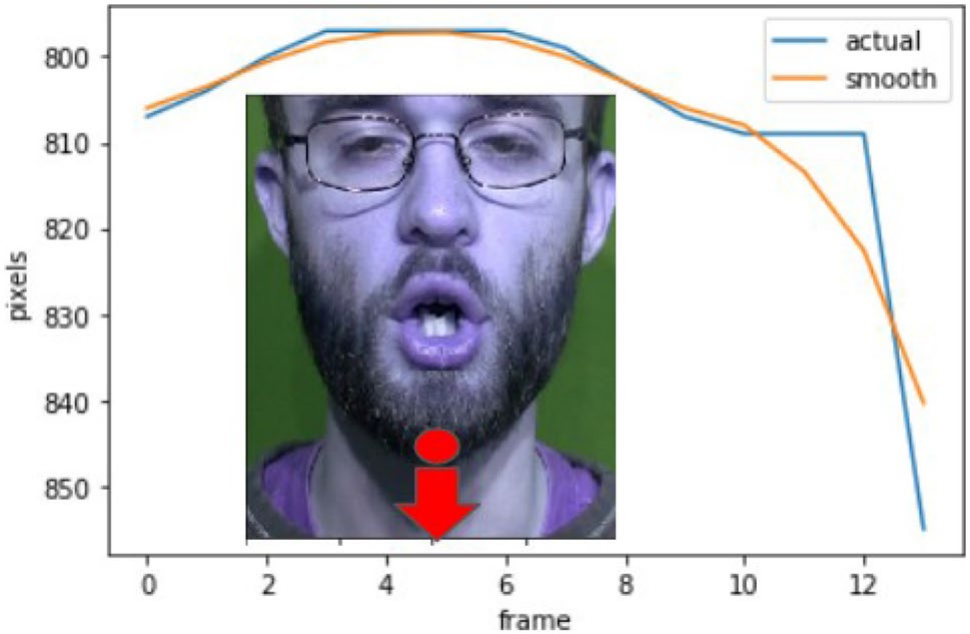
Trajectory of landmark number 9 (jaw lowering), as highlighted in the figure, is shown before and after temporal smoothing

The landmarks obtained from the SAN landmark detector are state of the art and generally smooth. However, in some cases, the extracted location of landmarks may deviate from their actual position on the face. To address this, we take advantage of the fact that, typically, only small changes in landmark locations occur between consecutive frames, i.e., temporally smooth without sudden changes to the landmark positions between frames. For example, in Fig. 3, landmark number 9 shows the resulting smooth trajectory when comparing across consecutive frames of a video. To improve the prediction of SAN in cases of temporal discontinuity of coordinate locations, we fit two degree 3 polynomial curves to each landmark, resulting in 136 curves depicting the motion 136 landmarks.

#### Spatial landmark smoothing

Once we have the destination landmarks, some landmarks may form a fold, depending on the magnitude of changes applied. For example, due to numerical error,^1^ lip landmarks may go above the nose landmarks. We know this is not possible so whenever a fold is detected, it is corrected by applying a Gaussian convolution to the lip region landmarks. This reduces the magnitude of change we apply to the lip region but in turn increases the smoothness.

### Displacement factor

**Fig. 4:**
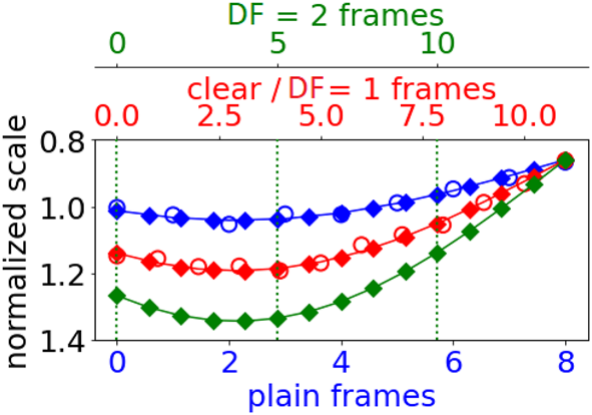
For the example word “could”, the hollow circles show the normalized position of the y component of the lower lip landmark across frames. Two degree 3 polynomial curves are drawn by fitting the plain (blue) and clear (red) landmark across frames. These curves can then be sampled for any number of sub-frame (here, 15 sub-frames indicated by diamonds on the curves). When the one-to-one displacements between the 15 plain (blue) and clear (red) sub-frame landmarks (diamonds) are added back to the plain (blue) landmarks, we obtain the 15 landmark points for the reconstructed landmarks at displacement factor (DF) 1 (red). When these displacements are doubled, we obtain the reconstructed landmarks for displacement factor 2 (green). The y-axis in this plot shows normalized scale (Sect. 3.2)

In the landmark manipulator, we also apply a displacement factor, which allows us to systematically vary the degree of clear speech modifications, as is illustrated in Fig. 4. A displacement factor (DF) is defined as a scalar weight applied to displacements and duration before adding them to the plain landmark frames to synthesize the clear landmark frames. A displacement factor of 1 corresponds to the movements and duration equivalent to clear speech, and a displacement factor greater than 1 would exaggerate the movements and duration even more than what is actually produced by the talker in clear speech. For example, suppose the displacement from the plain landmark frame to the clear landmark frame of the lower lip is 25% for the y-coordinate. If we choose a displacement factor of 2, we multiply this displacement by 2 and apply the change of 50% to the y-coordinate. The displacement factor is similarly applied in the duration domain, by multiplying the difference in the number of frames between plain and clear speech video (e.g., 3) by 2, and then adding it to the number of frames in the plain video (e.g., 9), resulting in (in case of the example in Fig. 4) 15 frames in the reconstructed clear video at displacement factor 2 ($3 \times 2 + 9 = 15$). Then, 15 points can be sampled from each of the 136 curves (Sect. 3.5).

### Image generator

To synthesize a clear speech video, we pass the destination landmarks (defined in Algorithm 1; see Appendix 1) to the image generator. The image generator first performs temporal smoothing of the destination landmarks’ positions by fitting a degree 3 continuous polynomial curve to the temporal change in the position of each of the 68 landmarks (similar to temporal smoothing described in Sect. 3.5). This is done to rectify potential errors in the output of the landmark manipulator and as there should only be small changes in landmark positions between frames. The image generator also has access to the mid vowel face image and the landmark frame from this face image. All the destination landmark frames are then normalized to the mid vowel landmark frame (brought to the scale and orientation of this mid vowel landmark frame). Piece-wise affine spatial transforms are calculated from the mid vowel landmark frame to each of the destination landmark frames. These transforms are then used to warp the RGB channels of the face image to synthesize the novel clear speech video. Since the warping operation is a pixel-to-pixel mapping from the High Definition (HD) quality original image to the warped image, we are able to preserve the HD quality in our videos, which is a much desired feature for human testing of visual speech intelligibility since high visibility and visual acuity have been shown to benefit speechreading (Lander & Cheryl, 2013; Legault et al., 2010).

## Generated videos

Using the materials described in Sect. 2, a set of videos (video only) is generated. Videos from four talkers (2 male, 2 female) are selected to be modified while videos from the remaining 14 talkers are used to calculate the displacement vectors. From each of these four talkers, we further choose one plain speech video for each word. This creates a subset of 24 (4 talkers $\times$ 6 words) plain videos to be modified.

Two modification targets (same-talker, average [Sect. 3.4]) are used to modify these videos to the extent equivalent to the natural clear speech style (DF1). Further, the target “average” is examined using two additional displacement factors (DF2 and DF3). For the “same-talker” target and for the “average” target, a displacement vector corresponding to DF1 is applied to obtain 24 modified videos each. For the “average” target, the displacement vector is then separately multiplied by 2 and 3 to obtain 24 DF2 and 24 DF3 videos, respectively. In total, the number of generated videos adds up to 96 (24 for the “same-talker” target and 72 for the “average” target).

**Fig. 5:**
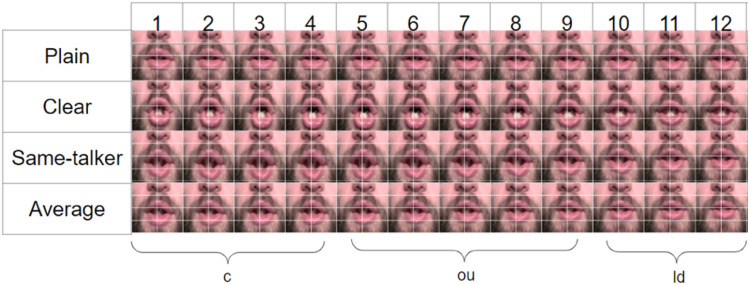
Natural plain and clear frames for the word “could” and modifications based on the “same-talker” and “average” targets

**Fig. 6:**
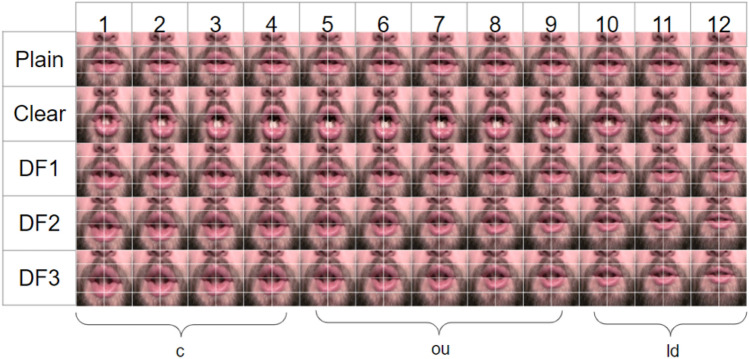
Natural plain and clear frames for the word “could” and modifications based on the “average” target at three displacement factor levels (DF1, DF2, DF3)

Figure 5 shows the modification results of an example word “could” for the “same-talker” and “average” targets. The results are visualized as a sequence of frames. Since changes in the mouth region are of interest, they are shown with an overlaid grid (in white) to show the extent of stretches. The first two rows show the plain and clear frames. Rows 3 and 4 visualize the modified frames obtained by using the “same talker” and “average” targets, respectively. Figure 6 shows the modification results of an example word “could” for three displacement factors: DF1, DF2, and DF3 for an “average” target. The first row in the figure is original plain video to be modified. The ground truth clear videos corresponding to these plain videos are shown in row 2. Rows 3, 4, and 5 show the modified frames for DF1, DF2, and DF3, respectively, obtained by using the “average” target. As shown in Fig. 6 (rows 3–5), stretching increases going from DF1 to DF3.

## Evaluation methodology

Once a set of videos was modified using the methods described above, the next step was to test if these visual modifications resulted in an increase in intelligibility. We assessed the intelligibility of the modified video-only utterances in two ways. The modified videos were presented to both AI Lip Reader (Sect. 5.1) and human participants (Sect. 5.2) for word identification.

### AI Lip Reader

First, we train the AI Lip Reader as a classification network. The problem statement presented to the Lip Reader is: Given this video-only, what word is being produced? The Lip Reader has six options: “keyed”, “kid”, “cod”, “cud”, “cooed”, and “could”. We use the Lip Reader as defined in Martinez et al. (2020). Deep learning, with its automatically learned features, has been shown to outperform conventional machine learning methods that rely on hand-crafted features in lip reading tasks (Fernandez-Lopez & Sukno, 2018; Pujari et al., 2021). In particular, deep learning based networks can achieve greater accuracy in feature extraction and classification, especially in vision based tasks (Fenghour et al., 2021), as is the case in the present study.

#### Model architecture

We use a deep learning based AI Lip Reader. The deep model architecture can be broken down into two main components. The first one is the ResNet-18 preceded by 3D convolution layer, which extracts feature maps. These features are then passed to the second major component, the Multi-scale Temporal Convolution Network (MS-TCN), which is used to exploit the temporal nature of the input being passed.

#### Data preparation

Each video sequence from the dataset has to be pre-processed before passing on to the AI Lip Reader network. In our experimentation, 29 frames are fed to the network. Since the frame rate of our videos is 29 frames/sec, this translates to the network accepting 1 s of video for each word. We have the timestamp of where the word starts and where it ends and use this to extract 29 sequential frames of the video. Any video that has more than 29 frames (i.e., a word is longer than 1 sec) is cut short by selecting frames at regular distance. For example, if the video is 2 s long, every alternate frame is selected so that we end up with only 29 frames. For each of the 29 frames, we follow the pre-processing steps suggested in Martinez et al. (2020):

1. Face detection and face alignment (as in Sect. 3.2).
2. Aligning each frame to a reference mean face shape. The mean shape comes from the training data of Multi-Task Cascaded Convolutional Neural Network (MTCNN). To obtain this mean face shape, all faces are selected and then the mean of each of the 68 landmarks is found across these faces. These 68 means then form the final reference mean face shape.
3. Cropping a fixed 96 × 96 pixels wide Region of Interest (ROI) from the aligned face image so that the mouth region is always roughly centered on the image crop.
4. Transform the cropped image to gray level.

#### Training the Lip Reader

We trained the Lip Reader on the original clear and plain videos without any audio (video only). This was done in a K-fold validation fashion where videos from the four talkers (discussed in Sect. 4) were used as test data while all the others made up the training data.

The AI Lip Reader achieved an accuracy of 83.0% in clear and 72.8% in plain speech videos. By comparison, the human perception data taken from Redmon et al. (2020), which contained the same set of videos with the same words and the same talkers, showed humans to have a lower accuracy, with 68.2% for the clear videos and 66.7% for plain videos.

These comparisons justify the use of this AI Lip Reader model since it outperforms humans in the classification task. We then employed this trained AI Lip Reader to test the generated videos and their corresponding original videos.

### Human evaluation of modified videos

We conducted two experiments to evaluate the intelligibility of the modified clear-speech videos (with no audio). Experiment 1 focused on the effects of modification target (same-talker, average). Experiment 2 addressed the effects of modification magnitude (displacement factors 1, 2, and 3). The two experiments were created using a custom version of jsPsych-6.1.0 and put on a JATOS server to be conducted online.

#### Experiment 1: Effects of modification target

*Stimuli* The stimuli in Experiment 1 include four sets of videos, each containing the six target words by 4 talkers: (1) 24 ground truth videos in plain speech, (2) 24 ground truth videos in clear speech, (3) 24 modified videos using same-talker target (DF1), and (4) 24 modified videos using average target (DF1).

*Participants* A total of 40 native English perceivers (aged 21-59), with normal hearing and vision were recruited via Amazon Mechanical Turk to participate in Experiment 1. The participants were randomly assigned to two groups: 20 (10 females, 10 males) were asked to identify the same-talker target videos, and the remaining 20 (7 females, 13 males) were tasked to identify the average target videos, both in addition to the ground truth plain and clear videos.

*Procedures* For both participant groups (same-talker, average), the stimuli were separated into three blocks, starting with a block of ground truth plain stimuli ($n = 24$), followed by ground truth clear and modified clear stimuli mixed together in two blocks ($n = 24$ each), with a short break in-between each of the blocks. The order of presentation of the stimuli was randomized within each block.

For each trial, one silent video clip of a talker speaking a word was presented. The perceiver was then asked to indicate (within 3 s) which word they perceived by clicking on one of the six target words (“keyed”, “kid”, “cod”, “cud”, “cooed”, and “could”) on the response screen. Each experiment lasted approximately 20 minutes, including practice and breaks.

#### Experiment 2: Effects of modification magnitude

*Stimuli* The stimuli in Experiment 2 included five sets of videos, each containing the six target words by four talkers: (1) 24 ground truth videos in plain speech, (2) 24 ground truth videos in clear speech, (3–5) 24 modified videos with each of the displacement factors 1, 2, and 3, all using the “average” target.

*Participants* Twenty native English perceivers (aged 21–60; 9 female, 11 male), with normal hearing and vision and who did not participate in Experiment 1, were recruited for Experiment 2 via Amazon Mechanical Turk.

*Procedures* The stimuli were presented in five blocks, starting with a block of ground truth plain stimuli ($n = 24$) followed by a block of ground truth clear stimuli ($n = 24$). The modified stimuli varying in displacement factor (DF1, 2, 3) were mixed together and presented in three subsequent blocks ($n = 24$ each). The ground truth clear stimuli were not presented along with the synthesized ones in the same block because the latter contain excessively modified tokens (e.g., DF3) which may be too contrastive with the natural stimuli and may thus affect perception. On each trial, after a silent target video was presented, the perceiver was to complete two tasks. In addition to identifying the target word (similar to Experiment 1), the participant was asked to rate how sure they were of their answer on a scale of 1 (not sure) to 5 (very sure). The addition of the rating task allowed evaluation of how confidently participants identify the synthesized visual stimuli, especially those with excessive modifications (e.g., DF3). Experiment 2 lasted about 30 min, on average.

## Results

The AI and human participant responses were compared directly for both Experiment 1 (effects of modification target: same-talker vs average talker) and Experiment 2 (effects of modification magnitude: DF1, DF2, DF3).

### Experiment 1: Effects of modification target

Effects of speech style and target were analyzed to examine how different targets affect intelligibility of the modified stimuli relative to natural ones. The dataset was submitted to a linear mixed-effects model using the ‘lmerTest’ package in R. The fixed effects include Target (same-talker, average), Responder (AI, human), Style (plain, clear, DF1), Vowel Tensity (tense, lax), and Talker Gender (male, female); the dependent variable being accuracy. A random effect was added on the intercept term to account for different words. After the model was finalized, a Type III Wald chi-square test was applied (using the Anova() function in the ‘car’ package) to assess the fixed effects including all the possible interaction terms. For significant interactions, subsequent post-hoc pairwise comparisons were conducted using the multivariate adjustment method (‘mvt’) in the ‘emmeans’ package. The generic model formula was:

$${\text{Word Identification Accuracy}}\,\sim \,{\text{Target}} \times {\text{Responder}} \times {\text{Style}} \times{\text{Tensity}} \times {\text{Talker Gender}} + \,\left( {1|{\text{Word}}} \right).$$

**Fig. 7:**
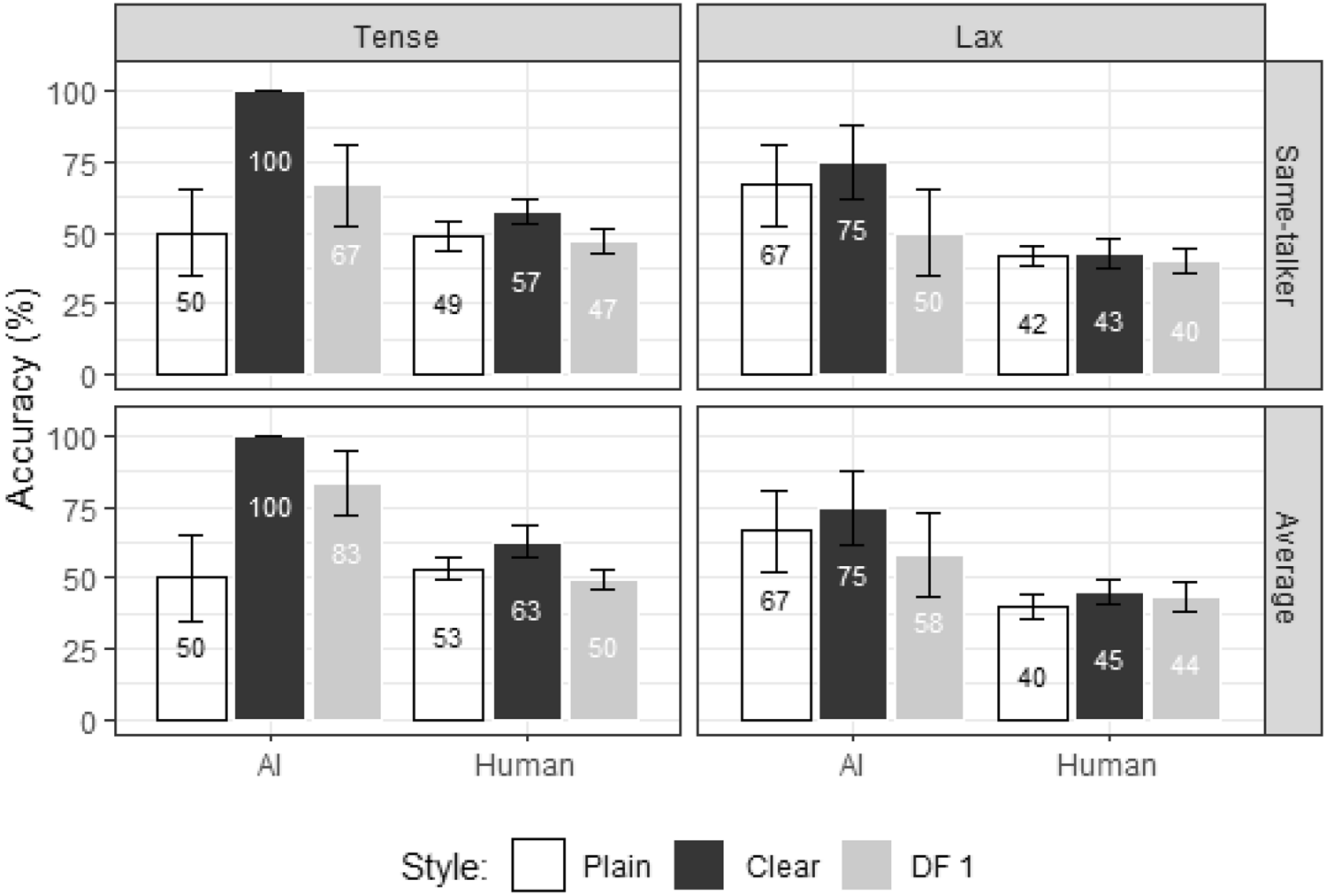
Accuracy and standard error for Experiment 1, broken down by Target (same-talker, average), Responder (AI, human), Style (plain, clear, DF1), and Tensity (tense, lax)

Model coefficient estimates are listed in Tables 2, 3, 4, 5, 6, and 7 in Appendix 2 (for the current model and subsequent models). Figure 7 displays the comparisons of identification.

Modeling results showed a significant main effect of Responder [*χ*^2^_(1)_ = 3.97, p = 0.046] with AI (70%) outperforming humans (48%). The model showed no significant effect of target, although the mean values showed a general trend of higher accuracy for the target “average” (61%) compared to the target “same-talker” (57%). In addition, a significant interaction between Style and Tensity was observed [*χ*^2^_(2)_ = 12.52, p = 0.002]. Post-hoc pairwise comparisons revealed that, for the tense-vowel words, accuracy was higher in the clear (80%) than plain (51%) style [Clear—Plain = 30%, CI (11%, 48%), t = 4.35, p < 0.001]. Furthermore, there was also a significant interaction of Style, Tensity and Talker Gender [*χ*^2^_(1)_ = 6.01, p = 0.014]. Post-hoc pairwise comparisons revealed that only tense-vowel words produced by female talkers showed greater accuracy for clear (80.2%) than plain speech videos (39%) [Clear—Plain = 41%, CI (11%, 70%), t = 4.24, p = 0.001].

Motivated by the significant main effect of Responder, and the interaction between Style and Tensity, we built separate models for AI tense vowel and human tense vowel data:

$${\text{Word}}\,{\text{Identification}}\,{\text{Accuracy}}\,\sim \,{\text{Target}} \times {\text{Style}} \times {\text{Talker}}\,{\text{Gender}} + \left( {1|{\text{Word}}} \right).$$

**Fig. 8:**
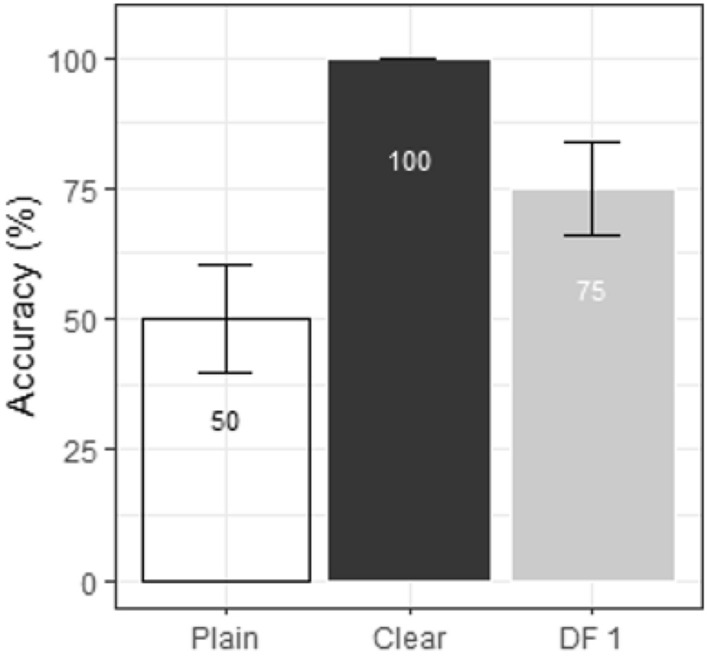
Word identification accuracy and 1 standard error for AI tense data in natural plain, natural clear and DF1 conditions in Experiment 1

As illustrated in Fig. 8, the AI tense condition showed a main effect of Style [*χ*^2^_(2)_ = 12.41, p = 0.002]. Post-hoc pairwise comparisons showed that clear words were more accurately identified (100%) than plain words (50%) [Clear—Plain = 50%, CI (26%, 74%), t = 5.08, p < 0.001], and than DF1 (75%) [Clear—DF1 = 25%, CI (1%, 49%), t = 2.54, p = 0.036]. In addition, the accuracy for DF1 was higher than that for Plain [DF1—Plain = 25%, CI (1%, 49%), t = 2.54, p = 0.036]. No other significant main effects or interactions were observed.

For human data, there were no significant main effects of style, nor were there significant main effects of any other factors or significant interactions.

### Experiment 2: Effects of modification magnitude

Effects of speech style and responder were analyzed through two sets of comparisons. The first analysis examined how participants’ word identification accuracy was affected by degree of modifications; and how AI and humans differed in their performance. The second analysis examined how confident participants were about their word choice. The dataset was submitted to a linear mixed-effects model using the ‘lmerTest’ package in R. Fixed effects were analyzed in the same fashion as described in Experiment 1 and with the same type of post-hoc comparisons. For the accuracy analysis, the fixed effects included Responder (AI, human), Style (plain, clear, DF1, DF2, DF3), Vowel Tensity (tense, lax), and Talker Gender (male, female). The confidence rating analysis included the same effects except Responder since we collected rating data from human participants only. The dependent variables for the two analyses were “identification accuracy” and “confidence rating score”, respectively. The confidence rating scores ranged from 1 to 5, where 1 refers to’unsure’ and 5 refers to’very sure’.

#### Accuracy

Participants’ identification accuracy in Experiment 2 was modeled using the following formula:

$${\text{Word Identification Accuracy}} \sim {\text{Responder}} \times {\text{Style}} \times {\text{Tensity}} \times {\text{Talker Gender}} + \left( {1|{\text{Word}}} \right).$$

**Fig. 9:**
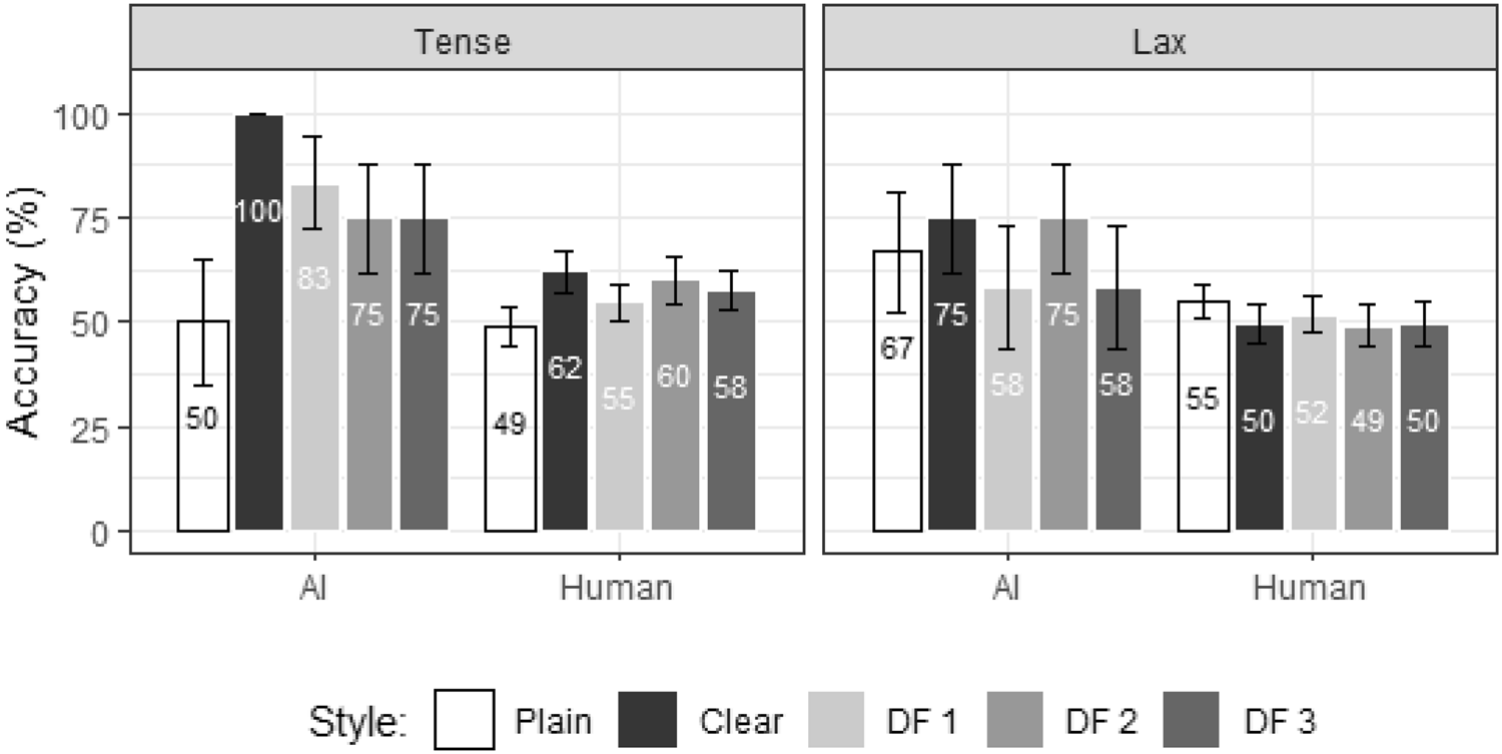
Accuracy and standard error for Experiment 2, shown for Responder (AI, human), Style (plain, clear, DF1, DF2, DF3), and Tensity (tense, lax)

Figure 9 displays the comparisons of identification accuracy.

Modeling results showed no significant main effects for Responder or Style. However, a significant interaction between Style and Tensity was observed [*χ*^2^_(4)_ = 12.9, p = 0.012]. Post-hoc pairwise comparisons revealed that, for the tense-vowel words, accuracy was higher in the clear (81%) than plain (49%) style [Clear-Plain = 32%, CI (5%, 58%), t = 3.29, p = 0.01]. Although there was no statistically significant difference, the mean accuracy for the tense-vowel words in all the three modified conditions tended to be higher than in the plain condition. Additionally, for AI, the accuracy of the DF1 condition tended to be higher than that of the DF2 and DF3 conditions.

#### Confidence rating

**Fig. 10:**
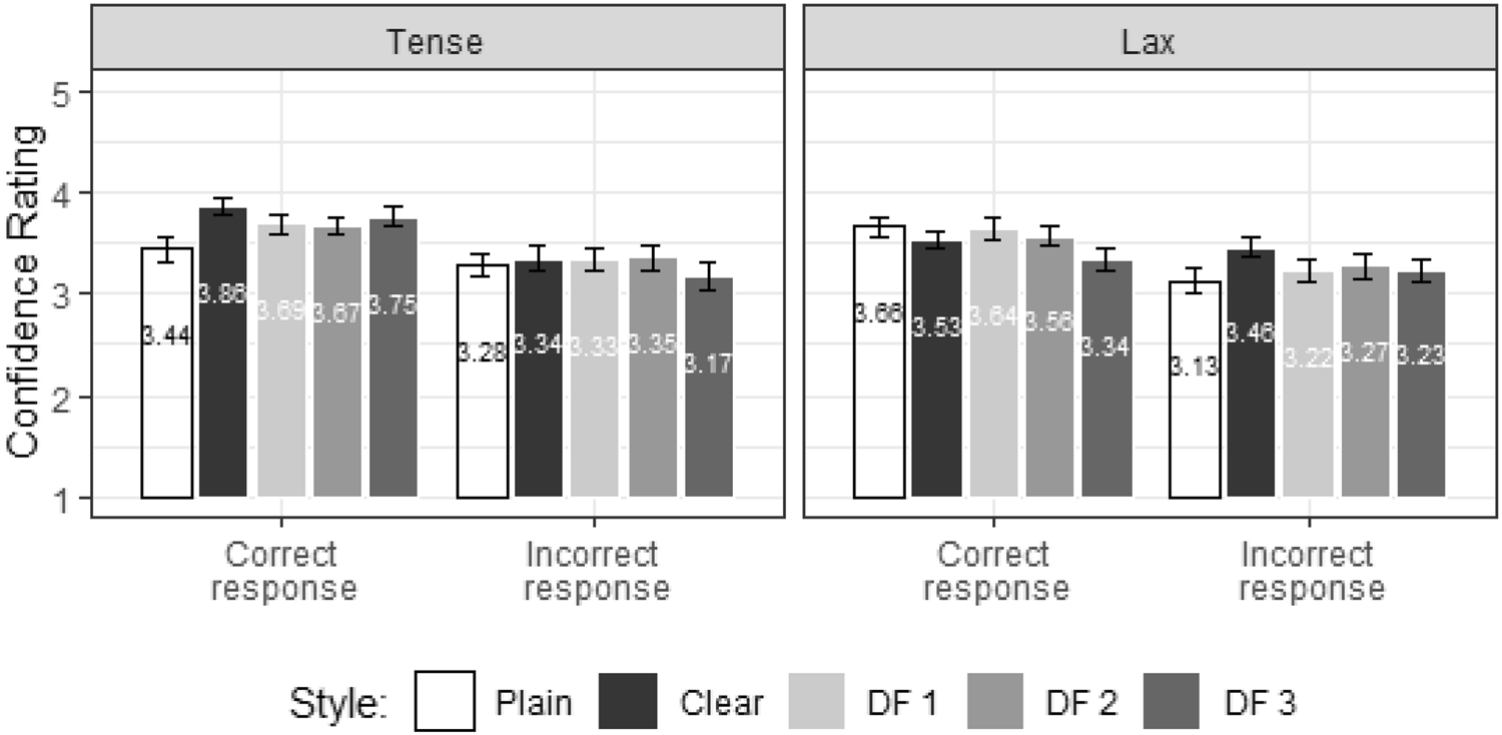
Confidence rating score (1 = not sure, 5 = very sure) and 1 standard error by human participants for Experiment 2, shown for Correctness (correct, incorrect), Style (plain, clear, DF1, DF2, DF3), and Tensity (tense, lax)

Confidence rating data were analyzed separately for correct responses and incorrect responses from the accuracy data in Sect. 6.2.1. Figure 10 displays the comparisons of identification confidence across conditions.

First, human participants’ correct responses in Experiment 2 were modeled using the following formula:

$${\text{Confidence Rating}} \sim {\text{ Style}} \times {\text{Tensity}} \times {\text{Talker Gender}} + \left( {1|{\text{Word}}} \right).$$

Confidence results for the “correct responses” showed a significant interaction between Style and Tensity [*χ*^2^_(4)_ = 11.06, p = 0.026]. Post-hoc pairwise comparisons revealed that, for the tense-vowel words, rating scores were higher in the clear (3.86) than plain (3.44) style [Clear—Plain = 0.432, CI (0.013, 0.851), t = 3.13, p = 0.038]. As shown in Fig. 10, for the “correct responses”, confidence rating scores of the modified stimuli (DF1, DF2, DF3) were intermediate to the natural clear and plain visual stimuli. The modified stimuli exhibited a trend of higher scores for the tense-vowel words than lax-vowel words across the three DFs, as well as a trend of a stepwise decrease in confidence for the lax-vowel words from DF1 to DF3.

To relate confidence ratings to the accuracy results, rating data for “correct responses” and “incorrect responses” were compared using the factor Correctness (Fig. 10), based on the following formula:

$${\text{Confidence Rating}} \sim {\text{Correctness}}\, \left( {{\text{correct}}, {\text{incorrect}}} \right) \times {\text{Style}} \times {\text{Tensity}} \times {\text{TalkerGender}} + \left( {1|{\text{Word}}} \right).$$

The results revealed a significant interaction of Correctness × Style × Tensity [*χ*^2^_(4)_ = 6.01, p = 0.026]. Post-hoc pairwise comparisons showed that confidence rating for “correct responses” was significantly or marginally higher than that for “incorrect responses” in the “clear, tense” (p = 0.064), “DF3, tense” (p = 0.05) and “plain, lax” (p = 0.03) conditions.

### Summary of results

First, for tense-vowel words in both Experiment 1 and Experiment 2, accuracy for natural clear videos was greater than that for natural plain.

Regarding the effects of clear-speech modification, in Experiment 1 AI tense-vowel data showed higher accuracy in the DF1 condition (modified clear-speech equivalent) than the plain condition. Experiment 2 exhibited the same trend.

With respect to the effects of modification target in Experiment 1, the results showed no statistically significant difference in intelligibility between the “same-talker” and “average” targets, although there was a trend suggesting that manipulations based on clear-speech features averaged across multiple talkers outperformed those based on talker-specific manipulations for both AI and human data.

For the effects of modification magnitude, Experiment 2 revealed a statistically non-significant trend for changes in intelligibility based on the extent to which the clear-speech features were added to the plain videos: tense-vowel modifications equivalent to natural clear speech (DF1) seemed to enhance AI (although not human) intelligibility to a greater degree than more excessive modifications (DF2 and DF3).

Experiment 2 further revealed that the confidence rating scores for human perception were generally closer to “very sure” (5) than “not sure” (1) on the rating scale, indicating that the participants were confident about their word identification. Moreover, rating scores of the modified stimuli (DF1, DF2, DF3) were comparable to the natural stimuli, suggesting the reliability of the identification results of the modified videos. Notably, for the modified stimuli, rating scores tended to be higher for the tense-vowel words than lax-vowel words, with confidence rating for the lax-vowel words decreasing as DF increased. Rating scores were also reliably higher for correctly identified compared to incorrectly identified tense-vowel words in the DF3 and clear conditions, whereas lax-vowel words only showed this difference in the plain condition. These rating patterns consistently suggest that modifications compatible with vowel features (e.g., stretching for tense vowels) were perceived with greater certainty than those deviating from inherent features (e.g., stretching for lax vowels).

Finally, AI was found to outperform human participants in Experiment 1, and the same trend held in Experiment 2.

## Discussion

### Effects of adding clear speech features

Previous research on naturally produced stimuli has shown that clear speech can improve human intelligibility in visual (facial) speech perception (Gagné et al., 1994, 2002; Helfer, 1997; Lander & Capek, 2013; Van Engen et al., 2014). More specifically, research from our team using automatic recognition of facial landmarks has shown that talkers change their speaking style and produce clear speech with exaggerated visual cues (Tang et al., 2015). Our further study linking articulation and perception (Redmon et al., 2020) reveals the advantage of clear-speech visual features in intelligibility for tense rather than lax vowels. Given these findings, we predicted that computationally modifying videos based on the vowel-intrinsic visual clear-speech features (cf. Sect. 3) would improve intelligibility of tense vowels for both AI and human responders.

The results from both Experiments 1 and 2 show that, consistent with the previous studies (Redmon et al., 2020), natural clear speech generally outperforms natural plain speech for tense-vowel words for both AI and humans. For the modified silent videos, the Experiment 1 results reveal improved intelligibility for AI in the DF1 condition, compared to the plain-speech condition in tense-vowel words, supporting our hypothesis. The DF1 condition implements a visual displacement factor that systematically varies the degree of visual modification equivalent to natural clear speech displacement. The Experiment 2 results show the same trend. These results suggest that adding clear-speech features to plain-speech videos does bridge the gap in intelligibility between plain and clear conditions, especially when the direction and characteristics of clear speech modifications are compatible with phoneme-inherent features such as in tense-vowel words rather than lax-vowel words (Redmon et al., 2020).

AI outperformed human perception of modified clear speech. Speech-reading research has consistently suggested that machines exceed humans in speech lip-reading (Martinez et al., 2020; Shillingford et al., 2018), in part because visual details associated with lip movements may be too subtle for humans to capture (Xiao et al., 2020). Moreover, in the current study, AI classification was based on a finite number of clear-speech features that the system was trained on, whereas human perceivers drew on the input signal and prior experience which could involve a vast variety of visual, non-speech, and even speech cues. While intelligibility by human perceivers was higher for the naturally produced clear compared to plain speech, the synthetically modified clear speech turned out not to be beneficial. The human results revealed no statistically significant difference between the modified and plain conditions.

Previous research has claimed that speech perception is the product of both signal-driven and signal-independent processes that incorporates speech-intrinsic and contextual information as well as physical variations in the signal (Kleinschmidt & Jaeger, 2015; Lindblom, 1990). Thus, the benefit of clear speech may be realized when all the information is available as a whole entity (both audio and visual enhancement as in natural clear speech) rather than by enhancing a number of known features only (as in the current synthetic visual speech). These findings suggest that future work may adopt unsupervised deep learning methods (with a sufficiently large speech sample size) which can capture a richer array of speech and visual-facial related visual articulatory movements in clear-speech productions. On the other hand, human perceivers can benefit from specific visual cues when their attention is directed to such cues (e.g., Chen & Massaro, 2008). The current AI results showing increased intelligibility due to clear-speech visual modifications may suggest that similar focused training could be applied to humans (e.g., hearing-impaired population, language learners) for intelligibility improvement.

### Effects of modification targets

Using the clear speech target for modifications, our assumption was that visual clear-speech features averaged across multiple talkers may overcome talker-specific idiosyncrasies and may thus more likely capture universal clear-speech features, compared to features extracted from a single talker. This assumption is based on previous findings on individual talker differences in clear-speech audio and video implementation. For some talkers, clear-speech modifications do not reach a threshold of contrast and may not provide clear-speech benefits in intelligibility (Ferguson & Kewley-Port, 2007; Smiljanić & Bradlow, 2011). Moreover, talker-specific visual features may interact with talker-universal phonetically relevant clear-speech cues and hinder intelligibility (Yakel et al., 2000) (e.g., the tense-lax distinctions).

The current results show no statistically significant difference in intelligibility for visual cues between the “average” and “same-talker” targets, despite a trend from the mean values suggesting that the “average” target might outperform the “same-talker” target condition. This trend is found to be uniform across all conditions. In addition, despite the observed difference for talker gender for tense-vowel words in clear speech, which is consistent with previous findings (Ferguson, 2004, 2012), this talker difference was not great enough to benefit the “same-talker” targets over the “average” targets. Our earlier modeling results (Redmon et al., 2020) indicate that both models with and without compensating talker variance captured the clear-speech advantage for tense vowels observed in the perceivers, although the talker-compensated model also showed a greater predicted signal-based (category-universal) clear-speech advantage. These patterns suggest that talker idiosyncrasies do not hinder extraction of category-defining visual clear-speech cues and may explain the lack of critical difference between the two types of targets. Such findings have practical implications for applications of our modification approach. In creating clear speech of a talker, we expect that using generic, talker-universal clear-speech features can achieve the same level of clear-speech advantage as using the talker’s own clear-speech features; that is, universal visual clear-speech features may be used to modify any talker’s speech style.

### Effects of modification magnitude

It has been claimed that clear-speech modifications must maintain “phonemic norms” and keep cue values within the intended category, so that phonemic categorical distinctions can be preserved while being enhanced (Moon & Lindblom, 1994; Ohala, 1995; Smiljanić et al., 2021). Excessive exaggerations or modifications incompatible with phoneme-intrinsic cues may obscure visual distinctiveness between sounds and inhibit intelligibility (Redmon et al., 2020). In this study, we hypothesized that changes in intelligibility may be based on the extent to which visual clear-speech features are added to the plain videos. Modifications equivalent to natural clear-speech may enhance intelligibility. However, excessive exaggerations or modifications incompatible with phoneme-intrinsic cues may obscure visual distinctiveness between sounds and inhibit intelligibility (Redmon et al., 2020).

The results show a significantly higher accuracy in natural clear than plain speech intelligibility for the tense- but not lax-vowel words, consistent with the above-mentioned finding that clear-speech modifications which violate the constraints characterizing lax vowels do not benefit intelligibility (Redmon et al., 2020). However, the three levels of modifications do not reveal any difference from either natural visual clear or visual plain speech, or among each other. Although in the AI tense-vowel condition, the mean accuracy in DF1 (83%) tend to be higher than DF2 (75%) and DF3 (75%) as well as natural plain (50%) (cf. Fig. 9). Likewise, the mean confidence rating results of the lax-vowel words show a decrease in confidence as DF increases (cf. Fig. 10). These patterns indicate a trend for decreased intelligibility in the more excessively modified stimulus conditions, in line with our prediction. It should also be noted that, despite not significantly outperforming the plain condition, the modified conditions do not significantly underperform the natural clear condition either. As claimed in previous research, the more static nature of tense (relative to lax) vowels dictates that tense vowels can be maintained long and stable in clear speech (Leung et al., 2016; Picheny et al., 1986; Smiljanić & Bradlow, 2009). The modified stimuli with more excessive stretching may still be classified as being within the canonical tense vowel categories. Subsequent work may employ separate modification strategies for tense- and lax-vowel words which are in line with the characteristics of tense and lax vowels, respectively.

## Conclusions and future work

This research is the first attempt to generate more intelligible visual speech videos by transferring video features from visual clear speech training data to plain videos of novel talkers. The contributions of the current approach and findings have four facets. First, our initial extraction successfully identifies relevant visual cues for video modifications across speech styles, and crucially, has been shown to achieve enhanced intelligibility for AI systems. Extracted visual speech cues for clear speech can be transferred to video to increase intelligibility. Second, this work suggests that universal talker-independent clear-speech features may be utilized to visually modify any talker’s speech style. Third, we introduce “Displacement factor” as a way of systematically scaling modifications between speech styles, with displacement similar to clear speech changes to more extreme enhancements. Finally, the generated videos are high definition in quality, making them good candidates for future human centric intelligibility and perceptual training studies.

Findings of this study also guide directions for future research. In order for the modified videos to significantly benefit human perceivers, further work may utilize deep learning methods to capture speech and related visual facial movements present in natural clear speech productions. Moreover, given the findings of the differences between tense and lax vowels, subsequent work may employ separate modification strategies on the basis of the characteristics and phonetic specification of different speech segments.

The research findings in this study are a first step toward the use of computerized visual speech modifications and synthesis of clear speech changes in a wide array of applications for speech intelligibility enhancement in adverse listening or visual environments, language learning and teaching, speech and hearing therapy, and the quickly expanding use of human–computer interfaces.

## Data Availability

The datasets generated and analysed during the current study, source code and supplementary material are available at https://github.com/ShubamSachdeva/visual-speech-enhancement

## Appendix 2

See Tables 2, 3, 4, 5, 6, and 7.

**Table 2:** Model summary of Word Identification Accuracy ~ Target × Responder × Style × Tensity × Talker Gender + (1|Word) for Experiment 1

	Estimate	Std. Error	df	t value	Pr( >|t|)
Intercept	0.83	0.14	213.79	6.07	< 0.001
Responder (human)	− 0.38	0.19	236.00	− 1.99	0.047
Target (same-talker)	0.00	0.19	236.00	0.00	1.000
Style (clear)	− 0.17	0.19	236.00	− 0.87	0.387
Style (average DF 1)	− 0.33	0.19	236.00	− 1.73	0.084
Tensity (tense)	− 0.50	0.19	213.79	− 2.57	0.011
Talker Gender (male)	− 0.33	0.19	236.00	− 1.73	0.084
Responder × Style (human, clear)	0.15	0.27	236.00	0.54	0.591
Responder × Style (human, average DF 1)	0.27	0.27	236.00	1.00	0.318
Target × Styleclear (same-talker, clear)	0.00	0.27	236.00	0.00	1.000
Target × Styleclear (same-talker, average DF 1)	− 0.17	0.27	236.00	− 0.61	0.541
Style × Tensity (clear, tense)	0.83	0.27	236.00	3.06	0.002
Style × Tensity (average DF 1, tense)	0.83	0.27	236.00	3.06	0.002
Style × Talker Gender (clear, male)	0.50	0.27	236.00	1.84	0.067
Style × Talker Gender (average DF 1, male)	0.50	0.27	236.00	1.84	0.067
Responder × Target × Style(human, same-talker, clear)	− 0.08	0.38	236.00	− 0.21	0.835
Responder × Target × Style(human, same-talker, average DF 1)	0.17	0.38	236.00	0.45	0.652
Responder × Style × Tensity (human, clear, tense)	− 0.66	0.38	236.00	− 1.71	0.088
Responder × Style × Tensity (human, average DF 1, tense)	− 0.74	0.38	236.00	− 1.92	0.057
Target × Style × Tensity (same-talker, clear, tense)	0.00	0.38	236.00	0.00	1.000
Target × Style × Tensity (same-talker, average DF 1, tense)	− 0.17	0.38	236.00	− 0.43	0.665
Responder × Style × Talker Gender (human, clear, male)	− 0.35	0.38	236.00	− 0.91	0.365
Responder × Style × Talker Gender (human, average DF 1, male)	− 0.30	0.38	236.00	− 0.77	0.440
Target × Style × Talker Gender (same-talker, clear, male)	0.00	0.38	236.00	0.00	1.000
Target × Style × Talker Gender (same-talker, average DF 1, male)	0.17	0.38	236.00	0.43	0.665
Style Lear Tensity Ense Talker Gender (M)	− 0.83	0.38	236.00	− 2.17	0.031
Style Tensity Ense Talker Gender (M)	− 0.83	0.38	236.00	− 2.17	0.031
Responder × Target × Style × Tensity (human, same-talker, average					
DF 1, tense)	0.07	0.54	236.00	0.13	0.899
Responder × Target × Style × Tensity (human, same-talker, clear,	0.16	0.54	236.00	0.29	0.774
Responder × Target × Style Talker Gender (human, same-talker,					
clear, male)	0.07	0.54	236.00	0.13	0.899
Responder × Target × Style Talker Gender (human, same-talker,					
average DF 1, male)	− 0.30	0.54	236.00	− 0.55	0.584
Responder × Style × Tensity × Talker Gender (human, clear, tense,	0.57	0.54	236.00	1.04	0.299
Responder × Style × Tensity × Talker Gender (human, average DF					
1, tense, male)	0.49	0.54	236.00	0.90	0.370
Target × Style × Tensity × Talker Gender (same-talker, clear, tense,	0.00	0.54	236.00	0.00	1.000
Target × Style × Tensity × Talker Gender (same-talker, average DF					
1, tense, male)	0.17	0.54	236.00	0.31	0.760
Responder × Target × Style × Tensity × Talker Gender (human,					
same-talker, clear, tense, male)	− 0.07	0.77	236.00	− 0.09	0.927
Responder × Target × Style × Tensity × Talker Gender (human,					
same-talker, average DF 1, tense, male)	0.01	0.77	236.00	0.01	0.992

For brevity, interactions that do not involve Style are left out

**Table 3:** Model summary of Word Identification Accuracy ~ Target × Style × Talker Gender + (1|Word) for Experiment 1, with “AI, tense” data only

	Estimate	Std. Error	df	t value	Pr( >|t|)
Intercept	0.33	0.19	7.67	1.76	0.117
Target (same-talker)	0.00	0.20	58.00	0.00	1.000
Style (clear)	0.67	0.20	58.00	3.38	0.001
Style (average DF 1)	0.50	0.20	58.00	2.54	0.014
Talker Gender (male)	0.33	0.20	58.00	1.69	0.096
Target × Style (same-talker, clear)	0.00	0.28	58.00	0.00	1.000
Target × Style (same-talker, average DF 1)	− 0.33	0.28	58.00	− 1.20	0.236
Target × Talker Gender (same-talker, male)	0.00	0.28	58.00	0.00	1.000
Style × Talker Gender (clear, male)	− 0.33	0.28	58.00	− 1.20	0.236
Style × Talker Gender (average DF 1, male)	− 0.33	0.28	58.00	− 1.20	0.236
Target × Style × Talker Gender (same-talker, clear, male)	0.00	0.39	58.00	0.00	1.000
Target × Style × Talker Gender (same-talker, average DF 1, male)	0.33	0.39	58.00	0.85	0.401

**Table 4:** Model summary of Word Identification Accuracy ~ Target × Style × Talker Gender + (1|Word) for Experiment 1, with “Human, tense” data only

	Estimate	Std. Error	df	t value	Pr( >|t|)
Intercept	0.45	0.07	9.39	6.04	< 0.001
Target (same-talker)	0.02	0.08	58.00	0.24	0.812
Style (clear)	0.15	0.08	58.00	1.92	0.060
Style (average DF 1)	0.04	0.08	58.00	0.43	0.665
Talker Gender (male)	0.17	0.08	58.00	2.16	0.035
Target × Style (same-talker, clear)	− 0.01	0.11	58.00	− 0.10	0.924
Target × Style (same-talker, average DF 1)	0.00	0.11	58.00	− 0.03	0.979
Target × Talker Gender (same-talker, male)	− 0.13	0.11	58.00	− 1.10	0.278
Style × Talker Gender (clear, male)	− 0.12	0.11	58.00	− 1.02	0.313
Style × Talker Gender (average DF 1, male)	− 0.14	0.11	58.00	− 1.25	0.216
Target × Style × Talker Gender (same-talker, clear, male)	0.00	0.16	58.00	− 0.01	0.992
Target × Style × Talker Gender (same-talker, average DF 1, male)	0.04	0.16	58.00	0.27	0.790

**Table 5:** Model summary of Word Identification Accuracy ~ Responder × Style × Tensity × Talker Gender + (1|Word) for Experiment 2

	Estimate	Std. Error	df	t value	Pr( >|t|)
Intercept	0.83	0.15	83.01	5.67	< 0.001
Responder (human)	− 0.22	0.19	196.00	− 1.13	0.262
Style (clear)	− 0.17	0.19	196.00	− 0.87	0.388
Style (average DF 1)	− 0.33	0.19	196.00	− 1.73	0.085
Style (average DF 2)	0.00	0.19	196.00	0.00	1.000
Style (average DF 3)	− 0.33	0.19	196.00	− 1.73	0.085
Tensity (tense)	− 0.50	0.21	83.01	− 2.40	0.018
Talker Gender (male)	− 0.33	0.19	196.00	− 1.73	0.085
Responder × Style (human, clear)	0.03	0.27	196.00	0.12	0.903
Responder × Style (human, average DF 1)	0.22	0.27	196.00	0.80	0.427
Responder × Style (human, average DF 2)	− 0.13	0.27	196.00	− 0.46	0.647
Responder × Style (human, average DF 3)	0.21	0.27	196.00	0.77	0.445
Style × Tensity (clear, tense)	0.83	0.27	196.00	3.06	0.003
Style × Tensity (average DF 1, Tense)	0.83	0.27	196.00	3.06	0.003
Style × Tensity (average DF 2, Tense)	0.50	0.27	196.00	1.84	0.068
Style × Tensity (average DF 3, Tense)	0.67	0.27	196.00	2.45	0.015
Style × Talker Gender (clear, male)	0.50	0.27	196.00	1.84	0.068
Style × Talker Gender (average DF 1, male)	0.50	0.27	196.00	1.84	0.068
Style × Talker Gender (average DF 2, male)	0.17	0.27	196.00	0.61	0.541
Style × Talker Gender (average DF 3, male)	0.50	0.27	196.00	1.84	0.068
Responder × Style × Tensity (human, clear, tense)	− 0.57	0.38	196.00	− 1.47	0.143
Responder × Style × Tensity (human, average DF 1, tense)	− 0.58	0.38	196.00	− 1.52	0.131
Responder × Style × Tensity (human, average DF 2, tense)	− 0.15	0.38	196.00	− 0.39	0.697
Responder × Style × Tensity (human, average DF 3, tense)	− 0.38	0.38	196.00	− 0.97	0.331
Responder × Style × Talker Gender (human, clear, male)	− 0.34	0.38	196.00	− 0.89	0.376
Responder × Style × Talker Gender (human, average DF 1, male)	− 0.33	0.38	196.00	− 0.87	0.388
Responder × Style × Talker Gender (human, average DF 2, male)	− 0.03	0.38	196.00	− 0.09	0.931
Responder × Style × Talker Gender (human, average DF 3, male)	− 0.36	0.38	196.00	− 0.93	0.353
Style × Tensity × Talker Gender (clear, tense, male)	− 0.83	0.38	196.00	− 2.17	0.032
Style × Tensity × Talker Gender (average DF 1, tense, male)	− 0.83	0.38	196.00	− 2.17	0.032
Style × Tensity × Talker Gender (average DF 2, tense, male)	− 0.67	0.38	196.00	− 1.73	0.085
Style × Tensity × Talker Gender (average DF 3, tense, male)	− 0.67	0.38	196.00	− 1.73	0.085
Responder × Style × Tensity × Talker Gender (human, clear,					
tense, male)	0.68	0.54	196.00	1.24	0.216
Responder × Style × Tensity × Talker Gender (human, average					
DF 1, tense, male)	0.52	0.54	196.00	0.95	0.344
Responder × Style × Tensity × Talker Gender (human, average					
DF 2, tense, male)	0.31	0.54	196.00	0.57	0.572
Responder × Style × Tensity × Talker Gender (human, average					
DF 3, tense, male)	0.37	0.54	196.00	0.67	0.501

For brevity, interactions that do not involve Style are left out

**Table 6:** Model summary of Confidence Rating ~ Style × Tensity × Talker Gender + (1|Word) for Experiment 2, with “correct response” data only

	Estimate	Std. Error	df	t value	Pr( >|t|)
Intercept	3.73	0.16	26.34	24.00	< 0.001
Style (clear)	− 0.24	0.20	1263.70	− 1.22	0.222
Style (average DF 1)	− 0.04	0.20	1263.14	− 0.19	0.847
Style (average DF 2)	− 0.08	0.19	1265.05	− 0.42	0.678
Style (average DF 3)	− 0.39	0.19	1264.56	− 1.99	0.047
Tensity (tense)	− 0.26	0.23	32.44	− 1.14	0.263
Talker Gender (male)	− 0.17	0.20	1264.18	− 0.89	0.374
Style × Tensity (clear, tense)	0.64	0.28	1263.79	2.30	0.022
Style × Tensity (average DF 1, tense)	0.27	0.28	1263.14	0.97	0.332
Style × Tensity (average DF 2, tense)	0.30	0.27	1264.22	1.09	0.276
Style × Tensity (average DF 3, tense)	0.82	0.28	1264.07	2.97	0.003
Style × Talker Gender (clear, male)	0.26	0.28	1263.22	0.91	0.364
Style × Talker Gender (average DF 1, male)	0.06	0.28	1263.11	0.23	0.822
Style × Talker Gender (average DF 2, male)	− 0.01	0.28	1264.70	− 0.02	0.982
Style × Talker Gender (average DF 3, male)	0.16	0.28	1263.38	0.55	0.581
Tensity × Talker Gender (tense, male)	0.07	0.28	1263.76	0.26	0.795
Style × Tensity × Talker Gender (clear, tense, male)	− 0.19	0.39	1263.18	− 0.49	0.622
Style × Tensity × Talker Gender (average DF 1, tense, male)	− 0.03	0.40	1263.31	− 0.08	0.934
Style × Tensity × Talker Gender (average DF 2, tense, male)	0.00	0.40	1264.18	0.01	0.995
Style × Tensity × Talker Gender (average DF 3, tense, male)	− 0.44	0.40	1263.23	− 1.10	0.272

**Table 7:** Model summary of Confidence Rating ~ Correctness × Style × Tensity × Talker Gender + (1|Word) for Experiment 2

	Estimate	Std. Error	df	t value	Pr( >|t|)
Intercept	2.98	0.18	174.99	16.16	< 0.001
correctness (correct)	0.75	0.22	2347.85	3.40	0.001
Style (clear)	0.52	0.23	2346.68	2.28	0.023
Style (average DF 1)	0.29	0.23	2346.06	1.26	0.209
Style (average DF 2)	0.22	0.23	2347.00	0.96	0.335
Style (average DF 3)	0.30	0.23	2346.66	1.32	0.188
Tensity (tense)	0.41	0.24	137.51	1.69	0.094
Talker Gender (male)	0.27	0.23	2347.04	1.19	0.235
Correctness × Style (correct, clear)	− 0.76	0.31	2347.37	− 2.47	0.013
Correctness × Style (correct, average DF 1)	− 0.33	0.31	2346.05	− 1.07	0.286
Correctness × Style (correct, average DF 2)	− 0.31	0.31	2348.60	− 1.01	0.315
Correctness × Style (correct, average DF 3)	− 0.70	0.31	2347.93	− 2.26	0.024
Style × Tensity (clear, tense)	− 0.42	0.32	2346.76	− 1.29	0.197
Style × Tensity (average DF 1, tense)	− 0.02	0.32	2346.36	− 0.06	0.950
Style × Tensity (average DF 2, tense)	0.11	0.34	2347.96	0.31	0.754
Style × Tensity (average DF 3, tense)	− 0.33	0.33	2346.73	− 1.01	0.311
Style × Talker Gender (clear, male)	− 0.36	0.31	2346.31	− 1.15	0.249
Style × Talker Gender (average DF 1, male)	− 0.37	0.32	2346.08	− 1.17	0.242
Style × Talker Gender (average DF 2, male)	− 0.12	0.31	2347.04	− 0.39	0.699
Style × Talker Gender (average DF 3, male)	− 0.36	0.31	2346.12	− 1.16	0.248
Correctness × Style × Tensity (correct, clear, tense)	1.06	0.44	2347.49	2.42	0.016
Correctness × Style × Tensity (correct, average DF 1, tense)	0.29	0.44	2346.36	0.67	0.502
Correctness × Style × Tensity (correct, average DF 2, tense)	0.20	0.44	2348.46	0.46	0.645
Correctness × Style × Tensity (correct, average DF 3, tense)	1.16	0.44	2347.60	2.65	0.008
Correctness × Style × Talker Gender (correct, clear, male)	0.62	0.43	2346.49	1.43	0.154
Correctness × Style × Talker Gender (correct, average DF 1,	0.44	0.43	2346.03	1.00	0.315
Correctness × Style × Talker Gender (correct, average DF 2,	0.12	0.43	2348.43	0.29	0.773
Correctness × Style × Talker Gender (correct, average DF 3,	0.52	0.43	2346.45	1.20	0.229
Style × Tensity × Talker Gender (clear, tense, male)	0.26	0.45	2346.43	0.57	0.567
Style × Tensity × Talker Gender (average DF 1, tense, male)	0.01	0.45	2346.67	0.03	0.975
Style × Tensity × Talker Gender (average DF 2, tense, male)	− 0.23	0.45	2348.00	− 0.51	0.613
Style × Tensity × Talker Gender (average DF 3, tense, male)	0.25	0.45	2346.22	0.56	0.576
Correctness × Style × Tensity × Talker Gender (correct, clear,					
tense, male)	− 0.46	0.62	2346.54	− 0.74	0.461
Correctness × Style × Tensity × Talker Gender (correct, average					
DF 1, tense, male)	− 0.05	0.61	2346.84	− 0.09	0.930
Correctness × Style × Tensity × Talker Gender (correct, average					
DF 2, tense, male)	0.22	0.62	2348.61	0.35	0.723
Correctness × Style × Tensity × Talker Gender (correct, average					
DF 3, tense, male)	− 0.69	0.61	2346.35	− 1.13	0.261

For brevity, interactions that do not involve Style are left out

## Footnotes

1 The error in the numerical computations caused by the finite precision of computers when representing floating-point or integer values.
